# The Tabanidae of the Mitaraka expedition, with an updated check list of French Guiana (Diptera)

**DOI:** 10.3897/zookeys.684.13197

**Published:** 2017-07-12

**Authors:** Tiago Kütter Krolow, Augusto Loureiro Henriques, Marc Pollet

**Affiliations:** 1 Universidade Federal do Tocantins (UFT), Coordenação de Ciências Biológicas, Cx. Postal 136, CEP 77500-000, Porto Nacional, TO, Brazil; 2 Instituto Nacional de Pesquisas da Amazônia (INPA), Coordenação de Biodiversidade, Cx. Postal 2223, CEP 69080-971, Manaus, AM, Brazil; 3 Research Institute for Nature and Forest (INBO), Kliniekstraat 25, B-1070 Brussels, Belgium; 4 Research Group Terrestrial Ecology (TEREC), Ghent University, K.L.Ledeganckstraat 35, B-9000 Ghent, Belgium; 5 Entomology Unit, Royal Belgian Institute for Natural Sciences (RBINS), Vautierstraat 29, B-1000 Brussels, Belgium

**Keywords:** Amazon basin, distribution, horse flies, list of species, Neotropics, new records

## Abstract

This paper documents the horse fly fauna collected in lowland rainforest in the southwesternmost part of French Guiana (Mitaraka). During this “Our Planet Revisited” survey nine tabanid species were recorded from French Guiana for the first time: *Chrysops ecuadorensis* Lutz, *C. incisus* Macquart, *Catachlorops amazonicus* Henriques & Gorayeb, *Chlorotabanus flagellatus* Krolow & Henriques, *Cryptoylus cauri* Stone, *Phaeotabanus phaeopterus* Fairchild, *Philipotabanus stigmaticalis* (Kröber), *Stypommisa captiroptera* (Kröber) and *Tabanus amapaensis* Fairchild. An updated check list of Tabanidae of French Guiana is presented, including 79 species and one unidentified *Chrysops*.

## Introduction

The horse flies (Diptera, Tabanidae) have a worldwide distribution with almost 4,400 valid species (Pape et al. 2011). The Neotropical region has the highest species richness with approximately 1,205 species (Henriques et al. 2012), about 28% of the global tabanid fauna.

In French Guiana tabanid diversity has only poorly been studied. Except for species described by e.g., Fabricius and Macquart in the 18^th^ and 19^th^ centuries, only few species have been recorded from this part of South America and the Kröber catalogue (1934) only lists 22 species. Subsequent species lists were provided by Floch (1955) and Floch and Fauran (1955). Fairchild (1970) extended the list of French Guiana to 38 species by compiling data from the literature (including original descriptions), Floch’s work, and by examining material from the Muséum National d´Histoire Naturelle (MNHN, Paris, France). In the second part of the same manuscript, through material received from A.S. Balachowsky, Fairchild described two new species and added eight new records, which further increased the number to 48 species. More recently Raymond et al. (1984) recorded another 15 species for the first time from French Guiana. Other significant inventories by Raymond (1986, 1987) investigating the efficiency of sampling methods also added new records and confirmed old ones. In contrast to the compiled number of species from the above-mentioned papers (63 spp.), in the most recent Neotropical catalogue merely 48 species were cited from French Guiana, with 35 restricted to French Guiana, and 13 with a wider Neotropical distribution (Coscarón and Papavero 2009).

In 2015, a biodiversity survey was conducted in the southwesternmost part of French Guiana (Pascal et al. 2015) that produced a substantial number of dipteran samples, including diverse Tabanidae (Pollet et al. 2015). The objective of the present paper is to document on the tabanid fauna encountered during the Mitaraka 2015 survey (French Guiana) and to present an updated check list of Tabanidae of French Guiana.

## Methods

**Figure 1.**
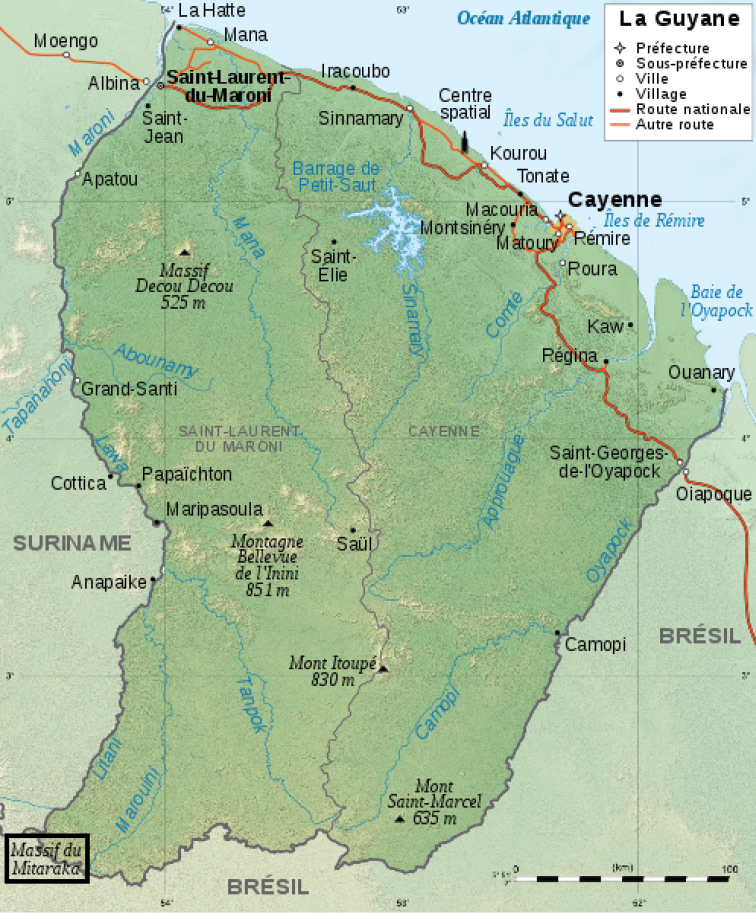
Map of French Guiana with indication of the investigated area (Mitaraka).

**Figures 2–5.**
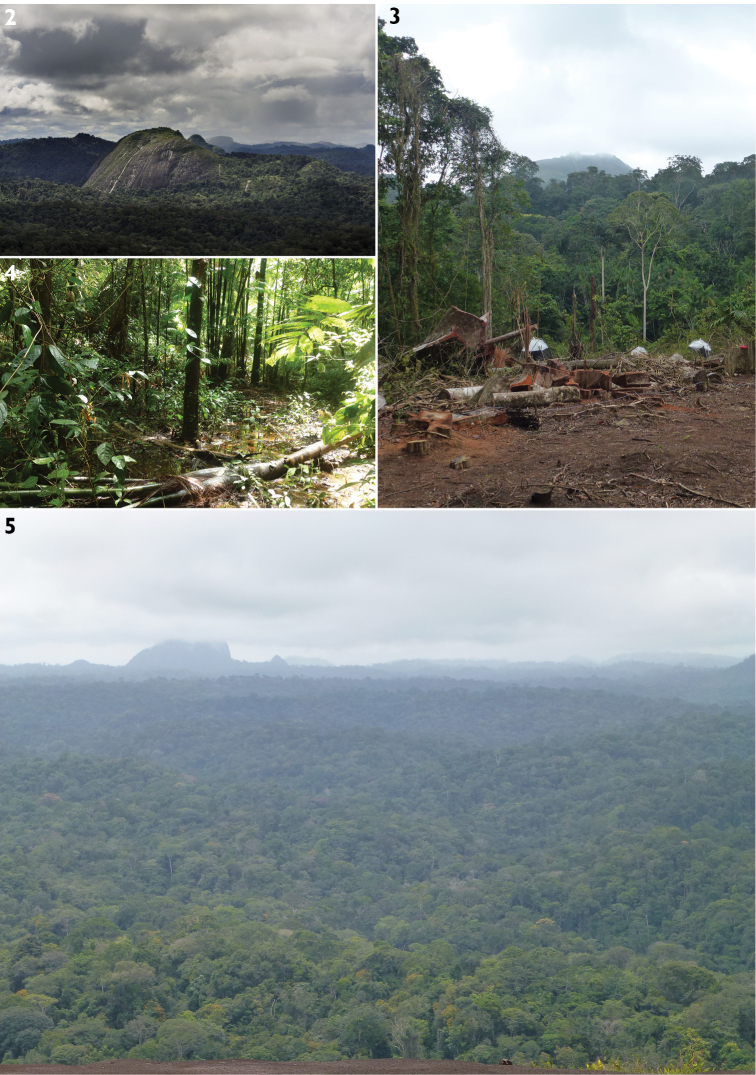
Investigated habitat types at Mitaraka **2** Inselberg Sommet-en-Cloche (photo Xavier Desmier) **3** drop zone (photo Marc Pollet) **4** river bed forest (photo Marc Pollet) **5** lowland rain forest of southern French Guiana (photo Marc Pollet).

In 2015 the “Our Planet Revisited” or “La Planète revisitée” Guyane 2014–2015 expedition, also known as the “Mitaraka 2015 survey”, was conducted in French Guiana (Pollet et al. 2014, Pascal et al. 2015). This was the 5^th^ edition of a large-scale biodiversity survey undertaken by the French Museum of Natural History in Paris and the NGO Pro-Natura international (both in France). Both organizations jointly run the “Our Planet Reviewed” programme which aims to rehabilitate taxonomical work that focuses on the largely neglected components of global biodiversity, i.e., invertebrates (both marine and terrestrial). Basic arthropod taxonomy and species discovery were at the heart of the survey, although forest ecology and biodiversity distribution modelling, nevertheless, were also part of the project. The expedition was conducted in the Mitaraka Mountains, a largely unknown and uninhabited area in the southwesternmost corner of French Guiana, directly bordering Surinam and Brazil (Fig. 1). It is part of the Tumuc Humac mountain chain, extending east in Amapa region and west in southern Surinam. The area consists primarily of tropical lowland rain forest with scattered inselbergs, isolated hills that stand above the forest plains (Figs 2–5).

**Figure 6.**
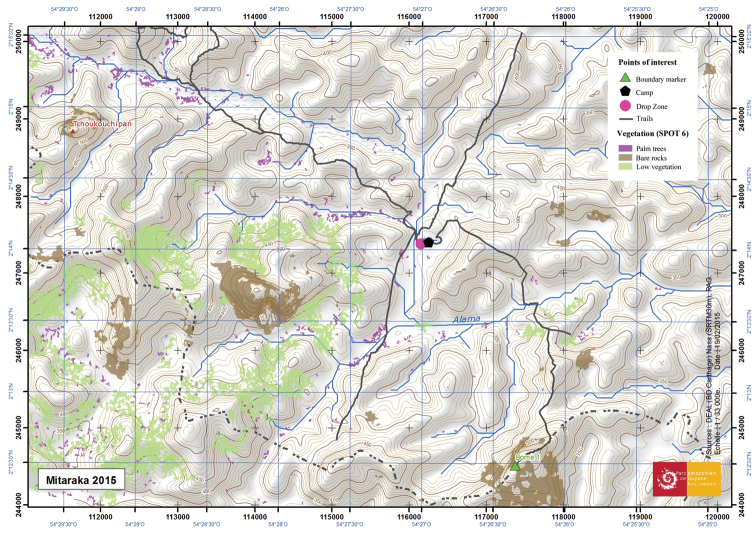
Mitaraka site map with four trails indicated (map by Maël Dewynter).

**Figures 7–10.**
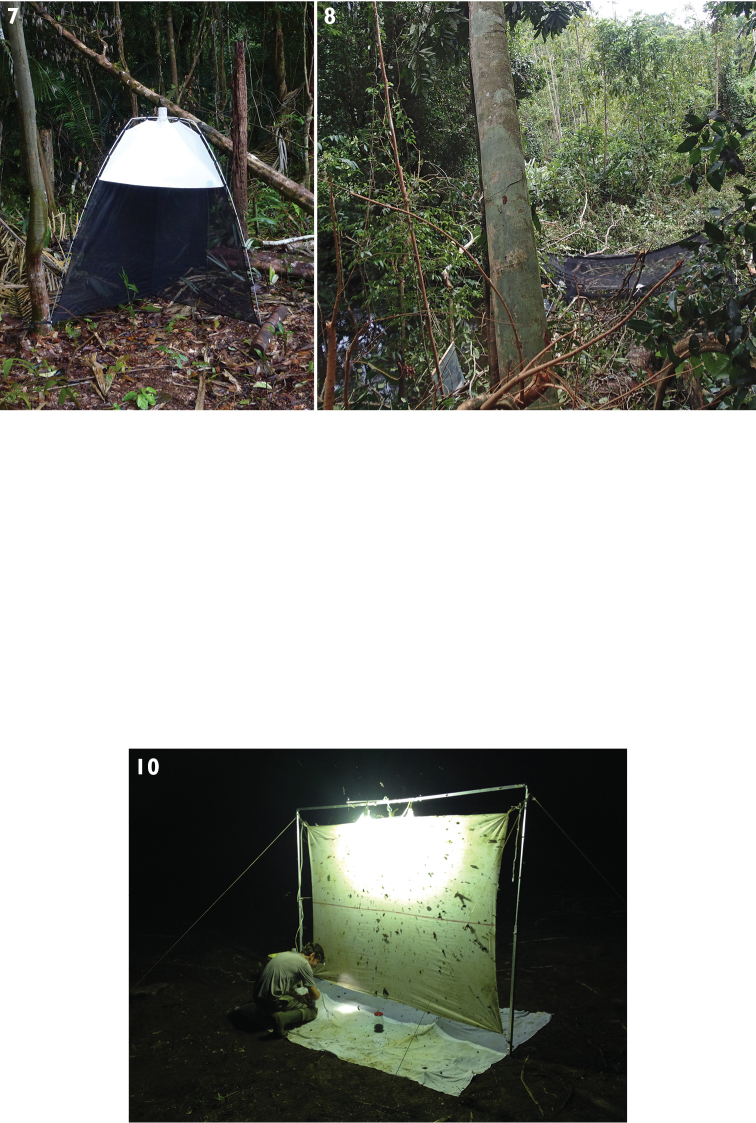
Collecting techniques applied during Mitaraka survey **7** SLAM (photo Marc Pollet), **8** 6m long Malaise trap (MT) (photo Julien Touroult) **9** flight intercept trap (FIT), with Eddy Porier (photo Julien Toroult) **10** light trap (LT), with Eddy Poirier (photo Marc Pollet).

From 22 February to 11 March 2015, a team of 32 researchers explored the area, including 12 invertebrate experts. During a second period (11 – 27 March), a second equal-sized team took over and a third smaller team returned to the site from 12 to 20 August 2015. MP was the coordinator of the collected Diptera, and was also the only Diptera worker actively involved in this survey. Invertebrate sampling was carried out near the base camp, on the drop zone (an area near the base camp that had been clear-cut entirely to allow helicopters to land) and, in particular, along four trails of approximately 3.5 km that started from the base camp in four different directions (Fig. 6). During the first period (22 February to 11 March 2015) more than 21 different collecting methods were applied, with a total of 401 traps operational within a perimeter of 1 km². This array consisted primarily of pan traps (n = 280), Charax butterfly traps (n = 50), square Malaise traps (SLAM) (n = 32), Flight Intercept Traps (FIT, n = 13) and Butterfly banana traps (n = 12), but also a light trap (Figs 7–10). In the second and third periods, pan traps were no longer included. A total of 217 invertebrate samples (often pooled yields of different traps of the same type) were examined, including 93 sweepnet samples, and 27 and 62 samples collected by SLAM and coloured pan traps (24 blue, 22 yellow and 16 white traps), respectively. As MP mainly focused on Dolichopodidae during active collecting, sweep net samples only rarely contained tabanids. Relevant metadata on the samples (e.g., exact locality and geographic coordinates, date or time period, collection method, and collector(s)) are provided in Appendix 1.

Non-pan trap samples were sorted to insect orders and families at the SEAG offices (http://insectafgseag.myspecies.info/fr), while pan trap samples were treated similarly at MP’s home lab. Dipteran subsamples (mostly per family) were subsequently disseminated among experts worldwide, in the case of Tabanidae to TKK and ALH. The identification of the tabanid species was conducted by ALH and TKK using taxonomical reviews and identification keys (Barretto 1950, Fairchild and Philip 1960, Fairchild 1976, Wilkerson and Fairchild 1982, Fairchild 1983, 1984, 1985, Gorayeb and Fairchild 1985, Fairchild and Wilkerson 1986, Burger 1996, Henriques and Gorayeb 1999, Henriques 2006, Krolow and Henriques 2010, Turcatel et al. 2010, Krolow et al. 2015), original descriptions, and direct comparison to reliably identified species from the Invertebrates Collection of the Instituto Nacional de Pesquisas da Amazônia, Manaus, Brazil (INPA) and the Entomological Collection of the Universidade Federal do Tocantins, Porto Nacional, Brazil (CEUFT). All collected material was stored in 70% alcohol during the expedition, being dry mounted on pins only about 11 months later in the laboratory. Preservation in alcohol usually affects the recognition of diagnostic features, which often no longer allows identification to species level.

In order to build an updated check list, species distribution records were compiled from the following literature: Fairchild (1976, 1983, 1984), Henriques and Gorayeb (1993), Henriques and Rafael (1993), Fairchild and Burger (1994), Henriques (1997), Coscarón and Papavero (2009), Krolow and Henriques (2010), Turcatel et al. (2010), Krolow et al. (2015), and Henriques (2016). Doubtful country records are indicated by “?”. Next to previously published records, all records from the Mitaraka 2015 survey are included in the check list. Each of these records is represented by the sample code and the number and gender of the collected specimens. Detailed information on the samples is given in Appendix 1. First records for French Guiana are explicitly indicated.

The specimens collected during the Mitaraka 2015 survey are deposited in the Muséum National d´Histoire Naturelle, Paris, France (**MNHN**), **CEUFT**, and **INPA**, according to an agreement between TKK and MNHN. Another acronym used in this paper is **AMNH**: American Museum of Natural History, New York, USA.

## Results

A total number of 255 tabanids of 24 species was collected during the Mitaraka 2015 survey. The subfamily Tabaninae is clearly the best represented with 19 species, followed by Chrysopsinae with three species, and Pangoniinae with two species. Of the 24 species only one belonging to *Chrysops* cannot be identified at a specific level. Female specimens were dominant in the samples, accounting for 233 specimens. Nineteen of the 22 males were collected at the light trap. The 6 m long Malaise trap that was installed over a river proved to be most productive, and collected nearly 2/5 of the specimens (see Table 1). Also SLAM traps, light traps, and flight intercept traps yielded at least 10 different species. In sharp contrast to this, neither blue nor yellow or white pan traps produced one single tabanid. In palm forests and forests along rivers, only *Bolbodimyia brunneipennis* Stone, *Dichelacera marginata* Macquart and *Pityocera cervus* (Wiedemann) were encountered. Fifteen different species were encountered on or near the drop zone and 16 species in the Malaise trap over the river. *D. marginata* seems to prefer humid sites near open water as only one specimen was collected on the drop zone compared to 15 in wet forests and 34 along the river.

**Table 1.** Overview of sampling methods that yielded Tabanidae during the Mitaraka 2015 survey.

Collecting methods*	**MT(6m)**	**LT**	**SLAM**	**FIT**	**SW**	**PVP**	**PVB**
Total number of examined samples	5	10	27	8	93	2	1
Tabanidae species (no. males + females)							
*Fidena auripes* (Ricardo)			2	3			
*Pityocera cervus* (Wiedemann)	4		9				
*Chrysops ecuadorensis* Lutz				1			
*Chrysops incisus* Macquart							1
*Chrysops* sp.			1	1			
*Bolbodimyia brunneipennis* Stone	4	1	8	1	1		
*Catachlorops amazonicus* Henriques & Gorayeb			1				
*Chlorotabanus flagellatus* Krolow & Henriques	1	2					
*Chlorotabanus inanis* (Fabricius)	3	5	1				
*Cryptoylus cauri* Stone	9	15	2				
*Diachlorus curvipes* (Fabricius)	2			5			
*Diachlorus fuscistigma* Lutz	2		1	1			
*Dichelacera damicornis* (Fabricius)	10	1	1			1	
*Dichelacera marginata* Macquart	34	1	18	1	3		
*Leucotabanus albovarius* (Walker)	1	6					
*Phaeotabanus phaeopterus* Fairchild	1						
*Philipotabanus stigmaticalis* (Kröber)		1	1				
*Stypommisa captiroptera* (Kröber)		6	1	1			
*Stypommisa modica* (Hine)	1						
*Tabanus amapaensis* Fairchild			1				
*Tabanus antarcticus* Linnaeus	1						
*Tabanus discus* Wiedemann	1						
*Tabanus occidentalis* Linnaeus	22	5		29			
*Tabanus trivittatus* Fabricius	2	15	1	1		1	
Number species	16	11	14	10	2	2	1
Number specimens	98	58	48	44	4	2	1

* MT(6m): 6m long Malaise trap, LT: light trap, SLAM: square Malaise trap, FIT: flight intercept trap, SW: sweep net, PVP: pink polytrap automatic light trap, PVB: blue polytrap automatic light trap.

This investigation revealed ten species recorded for the first time from French Guiana (see check list). After also screening previous records in the literature, an updated check list of 80 species of Tabanidae is presented here.

### List of species of Tabanidae from French Guiana

#### PANGONIINAE

##### SCIONINI

***Fidena analis* (Fabricius, 1805)**

**Records of French Guiana**: see Fairchild and Burger (1994).

**Distribution**: Guyana, French Guiana, Brazil (Amazonas).

***Fidena auripes* (Ricardo, 1900)**

Figure 11A

**Records of French Guiana**: see Fairchild (1970). **Examined material**: sample Mitaraka/219 (1♀ MNHNP); Mitaraka/224 (1♀ CEUFT; 1♀ INPA); Mitaraka/229 (1♀ CEUFT; 1♀ MNHNP).

**Distribution**: Guyana, Suriname, French Guiana, Brazil (Pará).

***Fidena aurulenta* Gorayeb, 1986**

**Records of French Guiana**: see Fairchild and Burger (1994).

**Distribution**: French Guiana, Brazil (Pará).

***Fidena mattogrossensis* (Lutz, 1912)**

**Records of French Guiana**: see Fairchild (1970), as *Fidena fulgifascies* Barretto, 1957.

**Distribution**: Guyana, Suriname, French Guiana, Brazil (Amazonas, Rondônia, Mato Grosso).

***Fidena pseudoaurimaculata* (Lutz, 1909)**

**Records of French Guiana**: see Fairchild (1970) and Henriques (2016).

**Distribution**: Venezuela, Guyana, Suriname, French Guiana, Brazil (Amazonas to Amapá, and Mato Grosso).

***Fidena schildi* (Hine, 1925)**

**Records of French Guiana**: see Fairchild (1970) and Henriques and Gorayeb (1993).

**Distribution**: Costa Rica to Colombia, French Guiana, Brazil (Roraima, Amazonas).

***Pityocera cervus* (Wiedemann, 1828)**

Figure 11B

**Records of French Guiana**: see Fairchild (1970), Henriques & Gorayeb (1993), Henriques (1997) and Krolow et al. (2015). **Examined material**: sample Mitaraka/150 (1♀ MNHNP); Mitaraka/186 (1♀ MNHNP); Mitaraka/189 (3♀ MNHNP); Mitaraka/199 (2♀ CEUFT); Mitaraka/202 (1♀ MNHNP); Mitaraka/207 (1♀ MNHNP); Mitaraka/208 (1♀ MNHNP); Mitaraka/213 (1♀ INPA); Mitaraka/229 (2♀ CEUFT).

**Distribution**: Colombia, Venezuela, Guyana, Suriname, Ecuador, French Guiana, Brazil (North), Peru, Bolivia.

#### CHRYSOPSINAE

##### CHRYSOPSINI

***Chrysops ecuadorensis* Lutz, 1909 – new to French Guiana**

Figure 11C

**Examined material**: sample Mitaraka/224 (1♀ CEUFT).

**Updated Distribution**: Ecuador, Peru (Madre de Dios), Guyana, French Guiana, Brazil (Pará).

***Chrysops formosus* Kröber, 1926**

**Records of French Guiana**: see Fairchild (1970).

**Distribution**: Trinidad, French Guiana, Brazil (Acre, Rondônia, Amazonas, Roraima, Pará, Amapá, Maranhão, Bahia).

***Chrysops incisus* Macquart, 1846 – new to French Guiana**

Figure 11D

**Examined material**: sample Mitaraka/227 (1♀ INPA).

**Updated Distribution**: Colombia, Guyana, Suriname, French Guiana, Brazil (Acre, Amazonas, Pará, Amapá, Maranhão), eastern Peru, Bolivia.

***Chrysops laetus* Fabricius, 1805**

**Records of French Guiana**: see Raymond et al. (1984).

**Distribution**: Colombia (Vaupés), Suriname, French Guiana, Brazil (Rondônia, Amazonas, Roraima, Pará, Amapá, Paraná, Rio Grande do Sul), ?Paraguay, ?Argentina (Misiones).

***Chrysops tristis* (Fabricius, 1798)**

**Records of French Guiana**: see Fabricius (1798) and Fairchild (1970).

**Distribution**: Trinidad, Venezuela, Guyana, Suriname, French Guiana, ?Brazil.

***Chrysops varians* Wiedemann, 1828**

**Records of French Guiana**: see Fairchild (1970).

**Distribution**: Panama, Trinidad, Colombia, Venezuela, Guyana, French Guiana, Ecuador, Peru, Brazil (Amapá to Rio Grande do Sul), Argentina (Misiones, Entre Ríos, Chaco), Paraguay.

***Chrysops variegatus* (De Geer, 1776)**

**Records of French Guiana**: see Fairchild (1970).

**Distribution**: Southern Mexico to Argentina (Misiones), West Indies.

***Chrysops venezuelensis* Kröber, 1925**

**Records of French Guiana**: see Raymond et al. (1984), as subspecies of *Chrysops variegatus*.

**Distribution**: Trinidad, Venezuela, Suriname, French Guiana, Brazil (Pará).

***Chrysops weberi* Bequaert, 1946**

**Records of French Guiana**: see Fairchild (1970).

**Distribution**: eastern Colombia, Venezuela, Guyana, French Guiana, Peru, Brazil (Rondônia, Amazonas).

***Chrysops* sp**.

Figure 11E

**Examined material**: sample Mitaraka/218 (1♀ CEUFT); Mitaraka/220 (1♀ MNHNP).

**Comment**: Two specimens of this morphotype were captured, but it was not possible to identify them with safety by the lack of recent taxonomic works of this genus.

##### RHINOMYZINI

***Betrequia ocellata* Oldroyd, 1970**

**Records of French Guiana**: see Raymond et al. (1984).

**Distribution**: eastern Colombia, French Guiana, Brazil (Amazonas, Pará, Ceará).

#### TABANINAE

##### DIACHLORINI

***Acanthocera gorayebi* Henriques & Rafael, 1992**

**Records of French Guiana**: see Henriques and Rafael (1999).

**Distribution**: Guyana, French Guiana, Peru, Brazil (Acre, Rondônia, Amazonas, Pará, Amapá, western Maranhão, Mato Grosso).

***Acanthocera marginalis* Walker, 1854**

**Records of French Guiana**: see Fairchild (1970) and Henriques and Rafael (1993).

**Distribution**: Colombia, Guyana, Suriname, French Guiana, Trinidad, Ecuador (Napo, Morona Santiago), Peru (Loreto), Brazil (Acre, Roraima, Amazonas, Pará, Amapá, Mato Grosso).

***Bolbodimyia brunneipennis* Stone, 1954**

Figure 11F

**Records of French Guiana**: according to Fairchild (1970), the specimen was erroneously identified by Surcouf (1921) as *Bolbodimyia bicolor* (Bigot) from the locality of Maroni. One female from Saint Laurent du Maroni is deposited at the AMNH (Henriques 2016). **Examined material**: sample Mitaraka/104 (1♀ MNHNP); Mitaraka/115 (1♂ CEUFT); Mitaraka/150 (2♀ MNHNP); Mitaraka/186 (2♀ CEUFT); Mitaraka/189 (2♀ MNHNP); Mitaraka/191 (1♀ MNHNP); Mitaraka/199 (1♀ MNHNP); Mitaraka/200 (1♀ MNHNP); Mitaraka/208 (1♀ MNHNP); Mitaraka/211 (1♀ MNHNP); Mitaraka/213 (1♀ INPA); Mitaraka/219 (1♀ CEUFT).

**Distribution**: Guyana, French Guiana, Brazil (Roraima, Pará, Amapá).

***Catachlorops amazonicus* Henriques & Gorayeb 1999** – **new to French Guiana**

Figure 11G

**Examined material**: sample Mitaraka/229 (1♀ INPA).

**Updated distribution**: French Guiana, Brazil (Amapá and Amazonas), Peru.

***Catachlorops balachowskyi* Fairchild, 1970**

**Records of French Guiana**: see Fairchild (1970).

**Distribution**: French Guiana.

***Catachlorops halteratus* Kröber, 1931**

**Records of French Guiana**: see Fairchild (1970).

**Distribution**: Guyana, Suriname, French Guiana, Peru (Loreto), Brazil (Rondônia, Amazonas, Roraima, Pará, Maranhão, Mato Grosso).

***Catachlorops rubiginosus*** (Summers, 1911)

**Records of French Guiana**: see Raymond et al. (1984) as *Catachlorops rubiginosa*.

**Distribution**: Guyana, French Guiana, Peru, Brazil (Amazonas, Pará, Mato Grosso).

***Catachlorops rufescens* (Fabricius, 1805)**

**Records of French Guiana**: see Fairchild (1970).

**Distribution**: Guyana, French Guiana, Brazil (Rondônia, Amazonas, Roraima, Pará, Maranhão, Mato Grosso).

***Chlorotabanus flagellatus* Krolow & Henriques, 2009 – new to French Guiana**

Figure 11H

**Examined material**: sample Mitaraka/100 (1♀ MNHNP); Mitaraka/102 (1♂ CEUFT); Mitaraka/186 (1♀ CEUFT).

**Updated distribution**: French Guiana, Brazil (Amazonas, Pará).

***Chlorotabanus inanis* (Fabricius, 1787)**

Figure 11I

**Records of French Guiana**: see Fairchild (1970), Krolow and Henriques (2010) and Henriques (2016). **Examined material**: sample Mitaraka/008 (1♂ MNHNP); Mitaraka/029 (1♂ CEUFT); Mitaraka/100 (1♀ MNHNP); Mitaraka/102 (1♀ CEUFT); Mitaraka/115 (1♀ MNHNP); Mitaraka/186 (1♀ MNHNP); Mitaraka/188 (2♀ MNHNP); Mitaraka/229 (1♀ MNHNP).

**Distribution**: Southern Mexico to southern Brazil.

***Chlorotabanus leucochlorus* Fairchild, 1961**

**Records of French Guiana**: see Fairchild (1970) and Krolow and Henriques (2010).

**Distribution**: Colombia, Venezuela, eastern Peru, Guyana, Suriname, French Guiana, Brazil (Amapá, Amazonas, Pará, Maranhão, Rondônia).

***Chlorotabanus leuconotus* Krolow & Henriques, 2010**

**Records of French Guiana**: see Krolow and Henriques (2010).

**Distribution**: Colombia, Guyana, French Guiana, Brazil (Roraima, Amazonas, Pará, Maranhão, Rondônia); Peru (Madre de Dios).

***Chlorotabanus mexicanus* (Linnaeus, 1758)**

**Records of French Guiana**: see Fairchild (1970), Krolow and Henriques (2010) and Henriques (2016).

**Distribution**: Mexico, Belize, Honduras, Nicaragua, Costa Rica, Panama, Colombia, Venezuela, Trinidad and Tobago, Suriname, French Guiana, Brazil (Pará), Ecuador.

***Cryptotylus cauri* Stone, 1944 – new to French Guiana**

Figure 11J

**Examined material**: sample Mitaraka/008 (1♀ MNHNP); Mitaraka/086 (1♀ CEUFT); Mitaraka/100 (9♀ MNHNP); Mitaraka/102 (1♀ CEUFT); Mitaraka/115 (3♀ CEUFT); Mitaraka/186 (2♀ MNHNP); Mitaraka/188 (1♀ MNHNP); Mitaraka/189 (4♀ MNHNP, 2♀ INPA); Mitaraka/229 (2♀ MNHNP).

**Updated Distribution**: Venezuela, Suriname, French Guiana.

***Cryptotylus unicolor* (Wiedemann, 1828)**

**Records of French Guiana**: see Fairchild (1970).

**Distribution**: Panama to Brazil (as far as Mato Grosso), Paraguay, Argentina (Chaco).

***Diachlorus bicinctus* (Fabricius, 1805)**

**Records of French Guiana**: see Raymond et al. (1984).

**Distribution**: Venezuela, Suriname, French Guiana, Trinidad, Peru, Bolivia, Brazil (Acre, Rondônia, Amazonas, Roraima, Pará, Amapá, Maranhão, Mato Grosso, Paraíba, Bahia).

***Diachlorus curvipes* (Fabricius, 1805)**

Figure 11K

**Records of French Guiana**: see Fairchild (1970). **Examined material**: sample Mitaraka/186 (1♀ MNHNP); Mitaraka/189 (1♀ CEUFT); Mitaraka/219 (3♀ MNHNP); Mitaraka/220 (1♀ MNHNP); Mitaraka/224 (1♀ CEUFT).

**Distribution**: Costa Rica, Panama to Suriname, French Guiana, eastern Peru, Bolivia and Brazil (Roraima, Pará, Amapá, Rondônia, Maranhão, Paraíba, Mato Grosso, ?Minas Gerais), Trinidad.

***Diachlorus fuscistigma* Lutz, 1913**

Figure 11L

**Records of French Guiana**: see Raymond et al. (1984). **Examined material**: sample Mitaraka/186 (1♀ MNHNP); Mitaraka/188 (1♀ CEUFT); Mitaraka/218 (1♀ MNHNP); Mitaraka/220 (1♀ MNHNP).

**Distribution**: Colombia, Suriname, French Guina, Ecuador, Peru (Loreto), Brazil (Acre, Rondônia, Amazonas, Roraima, Pará, Amapá, Bahia), Bolivia.

***Diachlorus scutellatus* (Macquart, 1838)**

**Records of French Guiana**: see Macquart (1838) and Fairchild (1970).

**Distribution**: Trinidad, Venezuela, Guyana, Suriname, French Guiana, Brazil (Amazonas, Pará).

***Dichelacera damicornis* (Fabricius, 1805)**

Figure 12A

**Records of French Guiana**: see Fairchild (1970) and Henriques and Gorayeb (1993). **Examined material**: sample Mitaraka/048 (1♀ MNHNP); Mitaraka/186 (5♀ MNHNP); Mitaraka/188 (3♀ MNHNP); Mitaraka/189 (3♀ CEUFT); Mitaraka/222 (1♀ CEUFT); Mitaraka/229 (1♀ MNHNP).

**Distribution**: ?Colombia, Venezuela to Brazil (Amazonas, Pará).

***Dichelacera marginata* Macquart, 1847**

Figure 12B

**Records of French Guiana**: see Macquart (1847), Fairchild (1970) and Henriques (2016). **Examined material**: sample Mitaraka/074 (2♀ MNHNP); Mitaraka/089 (1♀ MNHNP); Mitaraka/100 (1♀ MNHNP); Mitaraka/150 (10♀ CEUFT); Mitaraka/186 (17♀ MNHNP); Mitaraka/188 (6♀ MNHNP); Mitaraka/189 (10♀ MNHNP, 1♀ INPA); Mitaraka/191 (2♀ CEUFT); Mitaraka/192 (1♀ MNHNP); Mitaraka/195 (2♀ MNHNP); Mitaraka/207 (2♀ MNHNP); Mitaraka/229 (2♀ CEUFT).

**Distribution**: Nicaragua to northern Brazil and eastern Peru.

***Dichelacera t-nigrum* Fabricius, 1805**

**Records of French Guiana**: see Raymond et al. (1984).

**Distribution**: Venezuela and Guyana to Brazil (Pará).

***Lepiselaga crassipes* (Fabricius, 1805)**

**Records of French Guiana**: see Fairchild (1970).

**Distribution**: Mexico to northern Argentina (Formosa, Chaco, Salta, Tucumán, Santa Fé, Buenos Aires), Cuba, Jamaica, Hispaniola, Puerto Rico.

***Leucotabanus albovarius* (Walker, 1854)**

Figure 12C

**Records of French Guiana**: see Raymond et al. (1984). **Examined material**: sample Mitaraka/008 (1♀, 1♂ MNHNP); Mitaraka/100 (1♀, 1♂ MNHNP); Mitaraka/102 (1♀, 1♂ CEUFT); Mitaraka/189 (1♀ CEUFT).

**Distribution**: Guyana, Suriname, French Guiana, Ecuador (Napo, Orellana), Peru, Bolivia, Brazil (Acre, Rondônia, Amazonas, Roraima, Pará, Amapá).

***Leucotabanus exaestuans* (Linnaeus, 1758)**

**Records of French Guiana**: see Fairchild (1970).

**Distribution**: Mexico to Bolivia (Chapare) and Argentina (Salta, Chaco, Misiones), Trinidad.

***Leucotabanus janinae* Fairchild, 1970**

**Records of French Guiana**: see Fairchild (1970).

**Distribution**: Colombia, Suriname, French Guiana, Brazil (Amazonas, Pará, Amapá).

***Phaeotabanus cajennensis* (Fabricius, 1787)**

**Records of French Guiana**: see Fabricius (1787), Fairchild (1970).

**Distribution**: Trinidad to Colombia and French Guiana, Brazil (as far as São Paulo, Paraná) and Bolivia.

***Phaeotabanus fervens* (Linnaeus, 1758)**

**Records of French Guiana**: see Fairchild (1970).

**Distribution**: Trinidad and Venezuela to Argentina (Chaco).

***Phaeotabanus nigriflavus* (Kröber, 1930)**

**Records of French Guiana**: see Kröber (1930) and Fairchild (1970).

**Distribution**: Colombia, Venezuela, Guyana, Suriname, French Guiana, Trinidad, Ecuador, Peru, Brazil (Roraima, Amapá, Amazonas, Pará, Acre, Rondônia).

***Phaeotabanus phaeopterus* Fairchild, 1964 – new to French Guiana**

Figure 12D

**Examined material**: sample Mitaraka/188 (1♀ CEUFT).

**Updated distribution**: Panama (Darien), eastern Colombia, eastern Ecuador (Pichincha), French Guiana, Brazil (Roraima, Amazonas, Pará, Mato Grosso), eastern Peru.

***Philipotabanus stigmaticalis* (Kröber, 1931) – new to French Guiana**

Figure 12E

**Examined material**: sample Mitaraka/002 (1♀ MNHNP); Mitaraka/191 (1♀ CEUFT).

**Updated distribution**: Guyana, French Guiana, Brazil (Acre, Amazonas, Roraima, Pará, Amapá).

***Stibasoma festivum* (Wiedemann, 1828)**

**Records of French Guiana**: see Fairchild (1970) and Turcatel et al. (2010).

**Distribution**: French Guiana, Brazil (Acre, Amazonas, Pará, ?Mato Grosso), Argentina (Formosa).

***Stypommisa captiroptera* (Kröber, 1930) – new to French Guiana**

Figure 12F

**Examined material**: sample Mitaraka/100 (1♀, 1♂ INPA); Mitaraka/102 (3♂ CEUFT); Mitaraka/115 (1♂ MNHNP); Mitaraka/219 (1♀ CEUFT); Mitaraka/229 (1♂ INPA).

**Updated distribution**: Mexico to French Guiana, Brazil (Rondônia, Amazonas, Roraima, Pará), ?Paraguay.

***Stypommisa glandicolor* (Lutz, 1912)**

**Records of French Guiana**: see Fairchild (1970) and Henriques (2016).

**Distribution**: Costa Rica, Colombia, Suriname, French Guiana, Peru, Bolivia, Brazil (Acre, Rondônia, Amazonas, Pará, Amapá, Mato Grosso).

***Stypommisa modica* (Hine, 1920)**

Figure 12G

**Records of French Guiana**: see Henriques and Gorayeb (1993). **Examined material**: sample Mitaraka/188 (1♀ CEUFT).

**Distribution**: Guyana, French Guiana, Peru, Bolivia, Brazil (Acre, Rondônia, Amazonas, Pará).

***Stypommisa tantula* (Hine, 1920)**

**Records of French Guiana**: see Raymond et al. (1984).

**Distribution**: Guyana, French Guiana.

**Remarks**: this species was not recognized as *Stypommisa* by Fairchild and Wilkerson (1986), and neither transferred to another genus. For unclear reasons, it was omitted in the Fairchild and Burger catalog (1994), but listed as *Stypommisa* by Coscarón and Papavero (2009).

##### TABANINI

***Phorcotabanus cinereus* (Wiedemann, 1821)**

**Records of French Guiana**: see Fairchild (1970), as Stenotabanus (Phorcotabanus) cinereus.

**Distribution**: Colombia (Meta), Ecuador, Peru, French Guiana, Brazil (Amapá, Amazonas, Pará, Acre, Ceará), Bolivia, Argentina (Chaco, Salta).

***Poeciloderas quadripunctatus* (Fabricius, 1805)**

**Records of French Guiana**: see Raymond et al. (1984).

**Distribution**: Mexico to Argentina (Salta, Tucumán, Catamarca, Misiones, Entre Ríos, Buenos Aires).

***Tabanus amapaensis* Fairchild, 1961 – new to French Guiana**

Figure 12H

**Examined material**: sample Mitaraka/229 (1♀ CEUFT).

**Updated Distribution**: Suriname, French Guiana, Brazil (Amazonas, Pará, Amapá).

***Tabanus angustifrons* Macquart, 1848**

**Records of French Guiana**: see Macquart (1848), Fairchild (1984) and Raymond (1986).

**Distribution**: Colombia, Venezuela, French Guiana, Peru, Brazil (Rondônia, Amazonas, Roraima, Pará, Amapá, Mato Grosso).

***Tabanus antarcticus* Linnaeus, 1758**

Figure 12I

**Records of French Guiana**: see Fairchild (1970). **Examined material**: sample Mitaraka/186 (1♀ CEUFT).

**Distribution**: Trinidad, Venezuela, Suriname to Peru and Brazil (Amazon basin, Bahia).

***Tabanus callosus* Macquart, 1848**

**Records of French Guiana**: see Fairchild (1970).

**Distribution**: Colombia (Vaupés, Amazonas), Peru (Madre de Dios, Putumayo), Guyana, French Guiana, Brazil (Rondônia, Amazonas, Roraima, Pará, Amapá, ?Bahia).

***Tabanus casteetus* Fairchild, 1984**

**Records of French Guiana**: see Fairchild (1984), as *Tabanus testaceus* Macquart.

**Distribution**: Venezuela, French Guiana, Ecuador, Brazil (Amazonas).

***Tabanus crassicornis* Wiedemann, 1821**

**Records of French Guiana**: see Fairchild (1984).

**Distribution**: Colombia, Venezuela, Suriname, French Guiana, Brazil (Acre, Rondônia, Amazonas, Roraima, Pará, Amapá, Mato Grosso).

***Tabanus discifer* Walker, 1850**

**Records of French Guiana**: see Raymond (1986).

**Distribution**: Venezuela, Trinidad, Suriname, French Guiana, Brazil (Pará, Amazonas), Ecuador, Peru (Loreto), Bolivia.

***Tabanus discus* Wiedemann, 1828**

Figure 12J

**Records of French Guiana**: see Fairchild (1970). **Examined material**: sample Mitaraka/186 (1♀ CEUFT).

**Distribution**: Trinidad, ?Venezuela, Guyana, Suriname, French Guiana, Ecuador (Napo), Brazil (Acre, Rondônia, Amazonas, Roraima, Pará, Amapá, Mato Grosso).

***Tabanus fortis* Fairchild, 1961**

**Records of French Guiana**: see Fairchild (1970, 1984).

**Distribution**: Guyana, Suriname, French Guiana, Peru, Brazil (Amazonas, Pará, Amapá).

***Tabanus fumomarginatus* Hine, 1920**

**Records of French Guiana**: see Fairchild (1970).

**Distribution**: Suriname, French Guiana, Peru, Brazil (Amapá, Amazonas).

***Tabanus guyanensis* Macquart, 1846**

**Records of French Guiana**: see Macquart (1846) and Fairchild (1970, 1984).

**Distribution**: Colombia, French Guiana, eastern Ecuador, eastern Peru, Brazil (Amapá, Amazonas, Pará, Rondônia, Mato Grosso), eastern Bolivia.

***Tabanus importunus* Wiedemann, 1828**

**Records of French Guiana**: see Fairchild (1970).

**Distribution**: Panama, Guyana, French Guiana, Trinidad, Peru, Bolivia, to Brazil (Rio Grande do Sul), Paraguay.

***Tabanus kwatta* Fairchild, 1983**

**Records of French Guiana**: see Fairchild (1983).

**Distribution**: Venezuela, Suriname, French Guiana, Brazil (Pará).

***Tabanus nebulosus* De Geer, 1776**

**Records of French Guiana**: see Fairchild (1970).

**Distribution**: Belize, Trinidad, ?Barbados to Brazil (until Mato Grosso do sul), Argentina (Tucumán, Formosa, Corrientes, Santa Fé, Chaco).

***Tabanus occidentalis* Linnaeus, 1758**

Figure 12K

**Records of French Guiana**: in Fairchild (1970), as Tabanus dorsiger var. dorsovittatus Macquart. **Examined material**: sample Mitaraka/100 (2♀, 2♂ CEUFT); Mitaraka/115 (1♀ MNHNP); Mitaraka/186 (8♀ MNHNP); Mitaraka/188 (8♀ CEUFT); Mitaraka/189 (5♀, 1♂ MNHNP); Mitaraka/197 (1♀ MNHNP); Mitaraka/198 (2♀ MNHNP); Mitaraka/219 (13♀ MNHNP); Mitaraka/220 (12♀ MNHNP); Mitaraka/224 (1♀ MNHNP).

**Distribution**: Mexico to Argentina (Entre Ríos, Buenos Aires), Trinidad.

***Tabanus olivaceiventris* Macquart, 1847**

**Records of French Guiana**: see Bigot (1892), as *Tabanus pulverulentus*, and Fairchild (1970, 1984).

**Distribution**: Panama to Brazil (Pará, Amapá), Trinidad.

***Tabanus pellucidus* Fabricius, 1805**

**Records of French Guiana**: see Fairchild (1970, 1984).

**Distribution**: Colombia, Venezuela, Guyana, Suriname, French Guiana, Ecuador (Napo, Orellana, Pastaza), e. Peru, Brazil (Roraima, Amazonas, Pará, Amapá).

***Tabanus piceiventris* Rondani, 1848**

**Records of French Guiana**: see Fairchild (1970).

**Distribution**: Trinidad, Colombia, Venezuela, Guyana, Suriname, French Guiana, Ecuador (Napo, Orellana), Peru, Brazil (Acre, Rondônia, Amazonas, Roraima, Pará, Amapá, Maranhão, Tocantins), Bolivia.

***Tabanus pungens* Wiedemann, 1828**

**Records of French Guiana**: Raymond et al. (1984) and Raymond (1986).

**Distribution**: U.S.A. (Texas), Neotropics (except West Indies and Chile), Trinidad.

***Tabanus rubripes* Macquart, 1838**

**Records of French Guiana**: see Fairchild (1970).

**Distribution**: Panama to Paraguay.

***Tabanus tristichus* Fairchild, 1976**

**Records of French Guiana**: see Raymond (1986).

**Distribution**: Suriname, French Guiana, Brazil (Amapá, Pará).

***Tabanus trivittatus* Fabricius, 1805**

Figure 12L

**Records of French Guiana**: see Fairchild (1970). **Examined material**: sample Mitaraka/002 (1♀ MNHNP); Mitaraka/186 (1♂ MNHNP); Mitaraka/100 (2♀, 1♂ CEUFT); Mitaraka/102 (1♂ MNHNP); Mitaraka/115 (7♀, 2♂ MNHNP); Mitaraka/169 (1♀ MNHNP); Mitaraka/189 (2♀ MNHNP); Mitaraka/197 (1♀ MNHNP); Mitaraka/222 (1♂ MNHNP).

**Distribution**: ?Costa Rica, ?Panama, Colombia, Guyana, Suriname, French Guiana, Brazil (Rondônia, Amazonas, Roraima, Pará, Amapá, Maranhão, Tocantins).

**Tabanus vittiger ssp. guatemalanus Hine, 1906**

**Records of French Guiana**: see Fairchild (1970), as *Tabanus subsimilis guatemalanus* Hine.

**Distribution**: U.S.A. (Florida), Bahamas, West Indies (Cuba, Cayman Islands, Jamaica, Puerto Rico), southeastern Mexico to Suriname, French Guiana and northern Brazil.

***Tabanus wilkersoni* Fairchild, 1983**

**Records of French Guiana**: see Raymond (1986) and Henriques and Gorayeb (1993).

**Distribution**: e. Colombia, French Guiana, eastern Peru, Brazil (Amapá, Amazonas, Pará, Mato Grosso do Sul).

**Figure 11.**
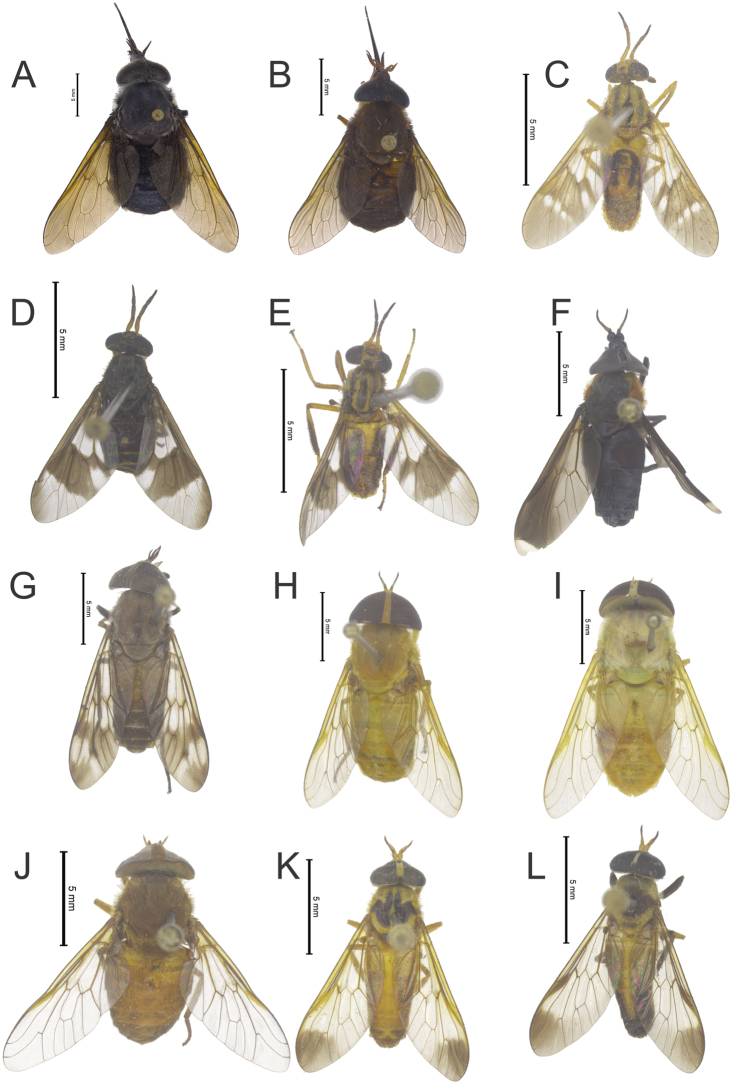
**A** *Fidena auripes* (Ricardo) **B** *Pityocera cervus* (Wiedemann) **C** *Chrysops ecuadorensis* Lutz **D** *Chrysops incisus* Macquart **E** *Chrysops* sp. **F** *Bolbodimyia brunneipennis* Stone **G** *Catachlorops amazonicus* Henriques & Gorayeb **H** *Chlorotabanus flagellatus* Krolow & Henriques **I** *Chlorotabanus inanis* (Fabricius) **J** *Cryptoylus cauri* Stone **K** *Diachlorus curvipes* (Fabricius) **L** *Diachlorus fuscistigma* Lutz. Photos by Augusto Henriques.

**Figure 12.**
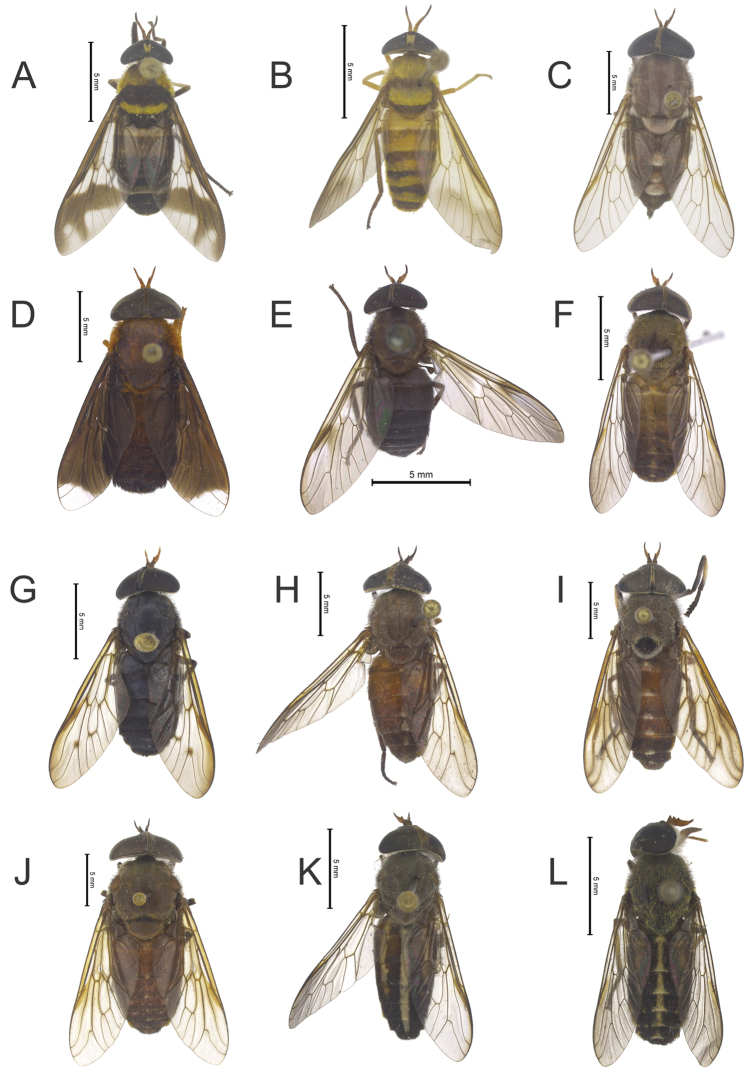
**A** *Dichelacera damicornis* (Fabricius) **B** *Dichelacera marginata* Macquart **C** *Leucotabanus albovarius* (Walker) **D** *Phaeotabanus phaeopterus* Fairchild **E** *Philipotabanus stigmaticalis* (Kröber) **F** *Stypommisa captiroptera* (Kröber) **G** *Stypommisa modica* (Hine) **H** *Tabanus amapaensis* Fairchild **I** *Tabanus antarcticus* Linnaeus **J** *Tabanus discus* Wiedemann **K** *Tabanus occidentalis* Linnaeus **L** *Tabanus trivittatus* Fabricius. Photos by Augusto Henriques.

### Record excluded from French Guiana

*Tabanus unipunctatus* (Bigot 1892) was cited from French Guiana by Fairchild (1970). However, in the Fairchild and Burger catalog (1994) the species distribution was corrected to: Mexico to western Colombia. Probably the 1970 Fairchild record refers to *T. fumomarginatus*.

## Discussion

French Guiana is part of the Guiana shield in northern Amazonia, bordering with Suriname in the west and Brazil (Amapá State) in the east, between the Maroni and Oiapoque rivers (Guitet et al. 2013). The Amazon rainforest covers more than 90% of this French department, while savannas and mangroves are present only along the coast (Guitet et al. 2014).

In their check list of insects of French Guiana, Brûlé and Touroult (2014) registered about 15,100 valid species names allocated in 20 orders and 322 families. According to the authors, Diptera is one of the poorest studied groups, with only 577 known species, including 6 endemic species, 50 species described from French Guiana, and 2 dubious records.

A high insect endemism in French Guiana is not very likely, because the country does not have strong geographical barriers with its neighbouring countries, Suriname and Brazil (Amapá) (Brûlé and Touroult 2014), and the same habitat types (or life zones) are present in each of these regions. This seems to be suggested by the observation that Suriname and Amapá share 49 and 42 species of Tabanidae (excluding the species with a large distribution) with French Guiana, respectively (Coscarón and Papavero 2009).

As expected, most species (76 sp.) observed in French Guiana belongs to the Amazonian tabanid fauna. Of its 80 species, 32 species have a large distribution in the Amazon basin, 30 species are shared by French Guiana with Suriname and/or Amapá state, and another 13 species with Guyana and/or Pará state. Three species have an even more extensive distribution range beyond French Guiana. Only one species might be endemic and another could not be identified, possibly a new species of *Chrysops*.

Currently, *Catachlorops balachowskyi* Fairchild seems endemic to French Guiana, while two other species, *Stypommisa tantula* (Hine) and *Fidena aurulenta* Gorayeb, are shared only with Guyana and Pará (Brazil), respectively.

The distribution records of Coscarón and Papavero (2009) were analysed, and it is estimated that approximately an additional 43 species have a high probability of occurring in French Guiana (Table 2). All estimated species have records from Suriname (11 spp.), Amapá (10 spp.), or both regions (2 spp.), or have a wide distribution in the Amazon region (20 spp.).

**Table 2.** List of Tabanidae known from neighbouring regions and expected to occur in French Guiana.

**N**°	**Species**	**Present occurrence**
1	*Esenbeckia osornoi* Fairchild, 1942	Suriname, Amapá
2	*Fidena loricornis* Kröber, 1931	Amapá
3	*Fidena nigripennis* (Guérin-Méneville, 1832)	Suriname
4	*Chrysops calogaster* Schiner, 1868	Amapá
5	*Chrysops guttipennis* Kröber, 1929	Suriname
6	*Chrysops leucospilus* Wiedemann, 1828	Amazon
7	*Acanthocera bequaerti* Fairchild & Aitken, 1960	Suriname
8	*Acanthocera fairchildi* Henriques & Rafael, 1992	Amazon
9	*Acanthocera polistiformis* Fairchild, 1961	Amapá
10	*Catachlorops difficilis* (Kröber), 1931	Amazon
11	*Catachlorops fumipennis* Kröber, 1931	Amazon
12	*Catachlorops testaceus* (Macquart, 1846)	Guyana, Amapá
13	*Diachlorus nuneztovari* Fairchild & Ortiz, 1955	Amazon
14	*Diachlorus pechumani aitkeni* Fairchild, 1972	Surinam
15	*Diachlorus podagricus* (Fabricius), 1805	Amazon
16	*Diachlorus xynus* Fairchild, 1972	Suriname
17	*Dichelacera bifacies* Walker, 1848	Amapá
18	*Dichelacera cervicornis* (Fabricius), 1805	Suriname, Amapá
19	*Dichelacera varia* (Wiedemann, 1828)	Amapá
20	*Eutabanus pictus* Kröber, 1930	Amapá
21	*Leucotabanus pauculus* Fairchild, 1951	Amazon
22	*Phaeotabanus innotescens* (Walker, 1854)	Suriname
23	*Phaeotabanus prasiniventris* (Kröber, 1929)	Amapá
24	*Philipotabanus pictus* Gorayeb & Rafael, 1984	Amazon
25	*Selasoma tibiale* (Fabricius, 1805)	Amazon
26	*Stenotabanus cretatus* Fairchild, 1961	Amapá
27	*Stenotabanus geijskesi* Fairchild, 1953	Suriname
28	*Stibasoma currani* Philip, 1943	Amazon
29	*Stibasoma flaviventris* (Macquart, 1848)	Amazon
30	*Stibasoma fulvohirtum* (Wiedemann, 1828)	Amazon
31	*Stypommisa prunicolor* (Lutz, 1912)	Amazon
32	*Stypommisa venosa* (Bigot, 1892)	Amazon
33	*Tabanus amazonensis* (Barretto, 1949)	Amazon
34	*Tabanus cicur* Fairchild, 1942	Amazon
35	*Tabanus claripennis* (Bigot, 1892)	Neotropical
36	*Tabanus curtus* Hine, 1920	Suriname
37	*Tabanus glaucus* Wiedemann, 1819	Amazon
38	*Tabanus macquarti* Schiner, 1868	Suriname
39	*Tabanus sannio* Fairchild, 1956	Amazon
40	*Tabanus secundus* Walker, 1848	Suriname
41	*Tabanus sorbillans* Wiedemann, 1828	Amazon
42	*Tabanus unimacula* Kröber, 1934	Suriname
43	*Tabanus xuthopogon* Fairchild, 1984	Amapá

With respect to the collecting methods, although interception traps (including Malaise traps and SLAM) are a passive method and without attractive power, they are among the most effective methods for capturing female tabanids, because the females are strong and frequent flyers, travelling great distances daily looking for a blood meal. The six meters Malaise trap is extremely effective for Tabanidae, and on some occasions several hundreds of specimens have been collected during one day (Gressitt and Gressitt 1962). According to Brown (2005), the Malaise trap method is especially effective to collect Neotropical Diptera, and Tabanidae seems to be one of 22 most abundant families in Malaise trap samples.

While the females are satisfactorily collected by interception traps, the males are rarely found in these traps, mainly because they are nectarivores, and thus do not need to travel far in search of warm-blooded hosts. As a result, male tabanids are also poorly represented in collections and even often unknown. Their rarity in interception traps might also be related to the effect of flowering periods, their preference to fly in higher tree strata or by their flight in restricted areas waiting for females to mate (Krolow et al. 2010). In contrast, males are commonly attracted to light, and the use of luminous attractant for collecting horse flies usually attracts much more males than females, usually of species with crepuscular habits (Frost 1951, Anthony 1960, Philip 1982, Fairchild 1986, Henriques and Rafael 1999).

Taking into account the large number of interception trap types employed during the Mitaraka (with only one operational light trap), female specimens were dominant in the samples as expected, accounting for 233 specimens, mostly collected by interception trap types, such as the 6m long Malaise trap (n = 98), SLAMs (n = 48), and flight intercept traps (n = 44). On the other hand, 19 of the 22 males were collected at the light trap, although, curiously, the trap collected more females than males (39 females vs 19 males) (see Table 1).

## Appendix 1

**Table 1A.** See Legend of Table 1 for explanation of abbreviations for collecting methods.

Sample id	Sample cd	Label
13882	MITARAKA/002	(FR-GU) Guyane Française, Mitaraka, MIT-DZ, 02°14’01.8”N, 54°27’01.0”W, 306 m, drop zone, 23.ii.2015, LT, leg. Julien Touroult (FR-GU/Mitaraka/2015) - sample code: MITARAKA/002 (sorted by Marc Pollet, 2015)
13888	MITARAKA/008	(FR-GU) Guyane Française, Mitaraka, MIT-DZ, 02°14’01.8”N, 54°27’01.0”W, 306 m, drop zone, 25.ii.2015, LT, leg. Marc Pollet (FR-GU/Mitaraka/2015) - sample code: MITARAKA/008 (sorted by Marc Pollet, 2015)
13909	MITARAKA/029	(FR-GU) Guyane Française, Mitaraka, MIT-DZ, 02°14’01.8”N, 54°27’01.0”W, 306 m, drop zone, 28.ii.2015, LT, leg. Marc Pollet (FR-GU/Mitaraka/2015) - sample code: MITARAKA/029 (sorted by Marc Pollet, 2015)
13928	MITARAKA/048	(FR-GU) Guyane Française, Mitaraka, MIT-DZ, 02°14’01.8”N, 54°27’01.0”W, 306 m, drop zone, 2.iii.2015, LT, leg. Jean-Hervé Yvinec (FR-GU/Mitaraka/2015) - sample code: MITARAKA/048 (sorted by Marc Pollet, 2015)
13954	MITARAKA/074	(FR-GU) Guyane Française, Mitaraka, MIT-A-RBF1, 02°14’11.4”N, 54°27’07.0”W, 306 m, on vegetation along muddy trail and in swamp, 6.iii.2015, SW, leg. Marc Pollet (FR-GU/Mitaraka/2015) - sample code: MITARAKA/074 (sorted by Marc Pollet, 2015)
13966	MITARAKA/086	(FR-GU) Guyane Française, Mitaraka, MIT-DZ, 02°14’01.8”N, 54°27’01.0”W, 306 m, drop zone, 7.iii.2015, LT, leg. Marc Pollet (FR-GU/Mitaraka/2015) - sample code: MITARAKA/086 (sorted by Marc Pollet, 2015)
13969	MITARAKA/089	(FR-GU) Guyane Française, Mitaraka, MIT-C-RBF2, 02°14’03.4”N, 54°26’53.0”W, 299 m, on leaf litter, muddy spots and vegetation along muddy trail, 8.iii.2015, SW, leg. Marc Pollet (FR-GU/Mitaraka/2015) - sample code: MITARAKA/089 (sorted by Marc Pollet, 2015)
13980	MITARAKA/100	(FR-GU) Guyane Française, Mitaraka, MIT-DZ, 02°14’01.8”N, 54°27’01.0”W, 306 m, drop zone, 9.iii.2015, LT, leg. Marc Pollet (FR-GU/Mitaraka/2015) - sample code: MITARAKA/100 (sorted by Marc Pollet, 2015)
13982	MITARAKA/102	(FR-GU) Guyane Française, Mitaraka, MIT-DZ, 02°14’01.8”N, 54°27’01.0”W, 306 m, drop zone, 9.iii.2015, LT, leg. Marc Pollet (FR-GU/Mitaraka/2015) - sample code: MITARAKA/102 (sorted by Marc Pollet, 2015)
13984	MITARAKA/104	(FR-GU) Guyane Française, Mitaraka, MIT-C-RBF2, 02°14’03.4”N, 54°26’53.0”W, 299 m, on vegetation along muddy trail and in swamp, 10.iii.2015, SW, leg. Marc Pollet (FR-GU/Mitaraka/2015) - sample code: MITARAKA/104 (sorted by Marc Pollet, 2015)
13995	MITARAKA/115	(FR-GU) Guyane Française, Mitaraka, MIT-DZ, 02°14’01.8”N, 54°27’01.0”W, 306 m, drop zone, 24.ii.2015-10.iii.2015, LT, leg. Julien Touroult (FR-GU/Mitaraka/2015) - sample code: MITARAKA/115 (sorted by Marc Pollet, 2015)
14030	MITARAKA/150	(FR-GU) Guyane Française, Mitaraka, MIT-A-RBF2, 02°14’12.5”N, 54°27’08.1”W, 287 m, tropical wet forest (bas fond), 27.ii.2015-10.iii.2015, SLAM, leg. Marc Pollet (FR-GU/Mitaraka/2015) - sample code: MITARAKA/150 (sorted by Marc Pollet, 2015)
14049	MITARAKA/169	(FR-GU) Guyane Française, Mitaraka, MIT-DZ1, 02°14’01.4”N, 54°27’00.2”W, 304 m, tropical moist forest (plateau-slope), 1.iii.2015-8.iii.2015, SLAM, leg. Marc Pollet (FR-GU/Mitaraka/2015) - sample code: MITARAKA/169 (sorted by Marc Pollet, 2015)
14064	MITARAKA/186	(FR-GU) Guyane Française, Mitaraka, nr MIT-A-RBF1, river, 1.iii.2015-7.iii.2015, MT(6m), leg. Julien Touroult & Eddy Poirier (FR-GU/Mitaraka/2015) - sample code: MITARAKA/186 (sorted by Marc Pollet, 2015)
14065	MITARAKA/188	(FR-GU) Guyane Française, Mitaraka, nr MIT-A-RBF1, river, 1.iii.2015, MT(6m), leg. Julien Touroult & Eddy Poirier (FR-GU/Mitaraka/2015) - sample code: MITARAKA/188 (sorted by Marc Pollet, 2015)
14066	MITARAKA/189	(FR-GU) Guyane Française, Mitaraka, nr MIT-A-RBF1, river, 25.iii.2015, MT(6m), leg. Julien Touroult & Eddy Poirier (FR-GU/Mitaraka/2015) - sample code: MITARAKA/189 (sorted by Marc Pollet, 2015)
14068	MITARAKA/191	(FR-GU) Guyane Française, Mitaraka, different sites nr base camp and along trails, tropical most forest (different sites), 14.iii.2015, SLAM, leg. Julien Touroult & Eddy Poirier (FR-GU/Mitaraka/2015) - sample code: MITARAKA/191 (sorted by Marc Pollet, 2015)
14069	MITARAKA/192	(FR-GU) Guyane Française, Mitaraka, different sites nr base camp and along trails, tropical most forest (different sites), 10.iii.2015-14.iii.2015, FIT, leg. Julien Touroult & Eddy Poirier (FR-GU/Mitaraka/2015) - sample code: MITARAKA/192 (sorted by Marc Pollet, 2015)
14072	MITARAKA/195	(FR-GU) Guyane Française, Mitaraka, different sites nr base camp and along trails, tropical most forest (different sites), 1.iii.2015-6.iii.2015, SLAM, leg. Julien Touroult & Eddy Poirier (FR-GU/Mitaraka/2015) - sample code: MITARAKA/195 (sorted by Marc Pollet, 2015)
14074	MITARAKA/197	(FR-GU) Guyane Française, Mitaraka, different sites nr base camp and along trails, tropical most forest (different sites), 20.iii.2015-25.iii.2015, FIT, leg. Julien Touroult & Eddy Poirier (FR-GU/Mitaraka/2015) - sample code: MITARAKA/197 (sorted by Marc Pollet, 2015)
14075	MITARAKA/198	(FR-GU) Guyane Française, Mitaraka, MIT-DZ, 02°14’01.8”N, 54°27’01.0”W, 306 m, tropical most forest (different sites) nr DZ, 6.iii.2015-10.iii.2015, FIT, leg. Julien Touroult & Eddy Poirier (FR-GU/Mitaraka/2015) - sample code: MITARAKA/198 (sorted by Marc Pollet, 2015)
14076	MITARAKA/199	(FR-GU) Guyane Française, Mitaraka, MIT-A-RBF2, 02°14’12.5”N, 54°27’08.1”W, 287 m, tropical wet forest (bas fond), 14.iii.2015-20.iii.2015, SLAM, leg. Julien Touroult & Eddy Poirier (FR-GU/Mitaraka/2015) - sample code: MITARAKA/199 (sorted by Marc Pollet, 2015)
14077	MITARAKA/200	(FR-GU) Guyane Française, Mitaraka, MIT-C-RBF2, 02°14’03.4”N, 54°26’53.0”W, 299 m, tropical wet forest (bas fond), 14.iii.2015-20.iii.2015, SLAM, leg. Julien Touroult & Eddy Poirier (FR-GU/Mitaraka/2015) - sample code: MITARAKA/200 (sorted by Marc Pollet, 2015)
14079	MITARAKA/202	(FR-GU) Guyane Française, Mitaraka, MIT-A-RBF2, 02°14’12.5”N, 54°27’08.1”W, 287 m, tropical wet forest (bas fond), 10.iii.2015-14.iii.2015, SLAM, leg. Julien Touroult & Eddy Poirier (FR-GU/Mitaraka/2015) - sample code: MITARAKA/202 (sorted by Marc Pollet, 2015)
14084	MITARAKA/207	(FR-GU) Guyane Française, Mitaraka, MIT-A-RBF2, 02°14’12.5”N, 54°27’08.1”W, 287 m, tropical wet forest (bas fond), 20.iii.2015-25.iii.2015, SLAM, leg. Julien Touroult & Eddy Poirier (FR-GU/Mitaraka/2015) - sample code: MITARAKA/207 (sorted by Marc Pollet, 2015)
14085	MITARAKA/208	(FR-GU) Guyane Française, Mitaraka, MIT-C-RBF2, 02°14’03.4”N, 54°26’53.0”W, 299 m, tropical wet forest (bas fond), 20.iii.2015-25.iii.2015, SLAM, leg. Julien Touroult & Eddy Poirier (FR-GU/Mitaraka/2015) - sample code: MITARAKA/208 (sorted by Marc Pollet, 2015)
14088	MITARAKA/211	(FR-GU) Guyane Française, Mitaraka, MIT-C-RBF2, 02°14’03.4”N, 54°26’53.0”W, 299 m, tropical wet forest (bas fond), 20.iii.2015-25.iii.2015, SLAM, leg. Julien Touroult & Eddy Poirier (FR-GU/Mitaraka/2015) - sample code: MITARAKA/211 (sorted by Marc Pollet, 2015)
14090	MITARAKA/213	(FR-GU) Guyane Française, Mitaraka, MIT-C-RBF2, 02°14’03.4”N, 54°26’53.0”W, 299 m, tropical wet forest (bas fond), 10.iii.2015-14.iii.2015, SLAM, leg. Julien Touroult & Eddy Poirier (FR-GU/Mitaraka/2015) - sample code: MITARAKA/213 (sorted by Marc Pollet, 2015)
14094	MITARAKA/218	(FR-GU) Guyane Française, Mitaraka, MIT-DZ, 02°14’01.8”N, 54°27’01.0”W, 306 m, tropical moist forest (plateau-slope - cleared), 1.iii.2015, SLAM, leg. Julien Touroult & Eddy Poirier (FR-GU/Mitaraka/2015) - sample code: MITARAKA/218 (sorted by Marc Pollet, 2015)
14095	MITARAKA/219	(FR-GU) Guyane Française, Mitaraka, MIT-DZ, 02°14’01.8”N, 54°27’01.0”W, 306 m, tropical moist forest (plateau-slope - cleared), 1.iii.2015, FIT, leg. Julien Touroult & Eddy Poirier (FR-GU/Mitaraka/2015) - sample code: MITARAKA/219 (sorted by Marc Pollet, 2015)
14096	MITARAKA/220	(FR-GU) Guyane Française, Mitaraka, MIT-DZ, 02°14’01.8”N, 54°27’01.0”W, 306 m, tropical moist forest (plateau-slope - cleared), 6.iii.2015, FIT, leg. Julien Touroult & Eddy Poirier (FR-GU/Mitaraka/2015) - sample code: MITARAKA/220 (sorted by Marc Pollet, 2015)
14098	MITARAKA/222	(FR-GU) Guyane Française, Mitaraka, different sites nr base camp and along trails, tropical most forest (different sites), 10.iii.2015, PVP, leg. Julien Touroult & Eddy Poirier (FR-GU/Mitaraka/2015) - sample code: MITARAKA/222 (sorted by Marc Pollet, 2015)
14100	MITARAKA/224	(FR-GU) Guyane Française, Mitaraka, different sites nr base camp and along trails, tropical most forest (different sites “sous bois”), 7.iii.2015, FIT, leg. Julien Touroult & Eddy Poirier (FR-GU/Mitaraka/2015) - sample code: MITARAKA/224 (sorted by Marc Pollet, 2015)
14103	MITARAKA/227	(FR-GU) Guyane Française, Mitaraka, different sites nr base camp and along trails, tropical most forest (different sites), 3.iii.2015, PVB, leg. Julien Touroult & Eddy Poirier (FR-GU/Mitaraka/2015) - sample code: MITARAKA/227 (sorted by Marc Pollet, 2015)
14304	MITARAKA/229	(FR-GU) Guyane Française, Mitaraka, different sites nr base camp and along trails, open / partially opened areas around base camp and drop zone, and in savane roche 2, 12.viii.2015-20.viii.2015, SLAM, leg. Pierre-Henri Dalens (FR-GU/Mitaraka/2015) - sample code: MITARAKA/229 (sorted by Marc Pollet, 2015)
